# Anti-oestrogen resistant human breast cancer cell lines are more sensitive towards treatment with the vitamin D analogue EB1089 than parent MCF-7 cells

**DOI:** 10.1054/bjoc.2000.1646

**Published:** 2001-03

**Authors:** S S Larsen, I Heiberg, A E Lykkesfeldt

**Affiliations:** Department of Tumor Endocrinology, Institute of Cancer Biology, Danish Cancer Society, Strandboulevarden 49, Copenhagen, DK-2100 Ø, Denmark

**Keywords:** MCF-7, anti-oestrogen resistance, ERα, Bcl-2, vitamin D analogue, EB1089

## Abstract

Most breast cancer patients treated with anti-oestrogens will eventually develop resistance towards treatment. Therefore it is important to find new therapeutic agents effective for treatment of patients relapsing on anti-oestrogen. The vitamin D analogue EB1089 (Seocalcitol^TM^) is a promising new agent for treatment of breast cancer patients with advanced disease, and in this study we show that two different anti-oestrogen-resistant human breast cancer cell lines are more sensitive towards treatment with EB1089, than the parent MCF-7 cell line. The two resistant cell lines both express a lower content of the anti-apoptotic protein Bcl-2, and we suggest that this may explain the higher sensitivity towards EB1089. The importance of Bcl-2 for response to EB1089 is supported by our observation that oestradiol abrogates the effect of EB1089 in cell lines which increase Bcl-2 in response to oestradiol treatment. Overall these results indicate that treatment with Seocalcitol^TM^may prove effective when patients become refractory to anti-oestrogen therapy, and that Bcl-2 may be used as a predictive marker. © 2001 Cancer Research Campaign http://www.bjcancer.com


British Journal of Cancer (2001) **84**(5), 686–690
© 2001 Cancer Research Campaign
doi: 10.1054/ bjoc.2000.1646, available online at http://www.idealibrary.com on IDEAL

http://www.bjcancer.com

### Anti-oestrogen resistant human breast cancer cell lines are more sensitive towards treatment with the vitamin D analogue EB1089 than parent MCF-7 cells

**SS Larsen, I Heiberg and AE Lykkesfeldt**

Department of Tumor Endocrinology, Institute of Cancer Biology, Danish Cancer Society, Strandboulevarden 49, DK-2100 Copenhagen Ø, Denmark

**Summary** Most breast cancer patients treated with anti-oestrogens will eventually develop resistance towards treatment. Therefore it is important to find new therapeutic agents effective for treatment of patients relapsing on anti-oestrogen. The vitamin D analogue EB1089 (Seocalcitol$^{TM}$) is a promising new agent for treatment of breast cancer patients with advanced disease, and in this study we show that two different anti-oestrogen-resistant human breast cancer cell lines are more sensitive towards treatment with EB1089, than the parent MCF-7 cell line. The two resistant cell lines both express a lower content of the anti-apoptotic protein Bcl-2, and we suggest that this may explain the higher sensitivity towards EB1089. The importance of Bcl-2 for response to EB1089 is supported by our observation that oestradiol abrogates the effect of EB1089 in cell lines which increase Bcl-2 in response to oestradiol treatment. Overall these results indicate that treatment with Seocalcitol$^{TM}$ may prove effective when patients become refractory to anti-oestrogen therapy, and that Bcl-2 may be used as a predictive marker. © 2001 Cancer Research Campaign http://www.bjcancer.com

**Keywords**: MCF-7; anti-oestrogen resistance; ERα; Bcl-2; vitamin D analogue, EB1089

Tamoxifen, a non-steroidal oestrogen antagonist, is the most widely used anti-oestrogen in endocrine treatment of advanced breast cancer. About 60% of the patients with ER-positive primary tumours respond to tamoxifen therapy for advanced disease (Osborne et al, 1980). Unfortunately, all patients will eventually develop resistance to tamoxifen treatment, but several patients will respond to second and third line of hormone therapy, such as progestins, aromatase inhibitors or the steroidal and pure antagonistic anti-oestrogen ICI 182 780 (Faslodex$^{TM}$) (Howell et al, 1995). Tamoxifen has both antagonistic and agonistic activities and the agonistic effects may be responsible for some cases of tamoxifen resistance (Howell et al, 1990). However, treatment with ICI 182 780 also result in outgrowth of resistant cells both in vitro, in cell cultures (Lykkesfeldt et al, 1995; Brünner et al, 1997), and in model studies in nude mice (Osborne et al, 1995). Development of resistance towards anti-oestrogens is thus a major clinical problem. It is therefore of extreme importance to obtain a detailed knowledge of anti-oestrogen resistance to further improve treatment of patients relapsing on anti-oestrogen treatment. The vitamin D analogue EB1089 is an interesting new and promising anti-cancer drug detailed reviewed in Hansen et al (2000). It has been shown to be highly antiproliferative and to induce apoptosis in breast cancer cells both in vitro and in vivo without causing hypocalcaemia (Mathiasen et al, 1993; Love et al, 1996; Xie et al, 1997, 1999; Gulliford et al, 1998; James et al, 1998; El Abdaimi et al, 1999; Mathiasen et al, 1999). EB1089 has also been shown to inhibit tumour growth of a newly established ICI 182 780 resistant MCF-7 subline in nude mice (Nolan et al, 1998). In this study, we have used two well characterized anti-oestrogen resistant cell lines, MCF-7/TAM$^{R}$-1 and MCF-7/182$^{R}$-6 (Lykkesfeldt and Briand, 1986; Lykkesfeldt and Sørensen, 1992; Wiseman et al, 1993; Lykkesfeldt et al, 1994, 1995; Larsen et al, 1997, 1999; Madsen et al, 1997; Jensen et al, 1999), and observed that these resistant cell lines responded to treatment with EB1089. In fact, they were more sensitive than the parent MCF-7 cells. The increased sensitivity to EB1089 treatment was found to be associated with a reduced level of the anti-apoptotic protein Bcl-2 in the resistant cells.

*Received 5 September 2000*
*Revised 15 November 2000*
*Accepted 17 November 2000*

*Correspondence to:* SS Larsen

### MATERIALS AND METHODS

### Cell cultures and growth experiments

The MCF-7 cell line was originally obtained from the Breast Cancer Task Force Cell Culture Bank, Mason Research Institute (Worcester, MA). The MCF-7 cells are routinely propagated in growth medium consisting of phenol red free DME/F12 (1:1) + 2.5 mM Glutamax (Life Technologies, Roskilde, Denmark) supplemented with 1% fetal calf serum (FCS) and 6 ng ml$^{-1}$ insulin (Novo-Nordic, Copenhagen, Denmark) (Lykkesfeldt et al, 1995). The tamoxifen resistant cell line MCF-7/TAM$^{R}$-1 and the ICI 182 780 resistant cell line MCF-7/182$^{R}$-6 were established as described earlier (Lykkesfeldt and Briand, 1986; Lykkesfeldt et al, 1995). Cultures used for growth experiments were seeded, $10^4$ cells/cm$^2$, in 2 cm$^2$ multiwell dishes (Nunc, Roskilde, Denmark). 2 days after seeding experimental media were added. Stock solution of $10^{-2}$ M oestradiol (Sigma, St Louis, MO) in 96% ethanol and stock solution of $4 \times 10^{-3}$ M EB1089 in 99% isopropanol (LEO Pharmaceuticals, Ballerup, Denmark) were stored in –20˚C freezer. For growth experiments EB1089 were further diluted in isopropanol. EB1089 and oestradiol were added to the experimental media at the time of change of media, which was every 2nd or 3rd day during the experiments. 4 wells were used for each cell number determination, which occurred at day 6 by counting in a Bürker-Türk chamber. Growth experiments were performed 3 times on independent cell lines.

### Western analysis

MCF-7, MCF-7/TAM$^{R}$-1 and MCF-7/182$^{R}$-6 cell lines were grown 1 week in control growth medium (1% FCS) before onset of the experiments. Cells were seeded in T25 flasks (Nunc) in control growth medium at a density of $2 \times 10^{5}$ cells/T25 flask. Experimental media were added 2 days after seeding, and medium was renewed every 2nd or 3rd day. After growth for 6 days in experimental medium, the cells were washed with PBS and harvested in RIPA-buffer (Larsen et al, 1999). 30 μg of total protein per sample (determined by Bio-Rad protein assay kit, Munich, Germany) were run on 15% SDS-PAGE gels under reducing conditions. The proteins were transferred to an Immobilon-P membrane (Millipore, Bedford, MA) by semi-dry electroblotting. Immunoassaying was done using a primary mouse monoclonal antibody against human Bcl-2 (Transduction Laboratories, Lexington, KY), a primary mouse monoclonal anti-human ERα antibody (1D5; DAKO, Glostrup, Denmark) and a secondary rabbit-anti-mouse horseradish peroxidase-conjugated antibody (P0260; DAKO). The blots were stripped and immuno-assayed with a primary mouse monoclonal antibody against human keratin 7 (kindly provided by Dr Jiri Lukas) as a control for differences in protein loading. The enhanced chemiluminescence (ECL$^{PLUS}$) detection system (Amersham Pharmacia Biotech, Hørsholm, Denmark) was used for visualization of the proteins according to the manufacturer's instructions. The blots were scanned and quantification was done with ImageQuant software (Amersham Pharmacia Biotech). The Western analysis was repeated 3 times on independent protein preparations and the results were reproducible.

### RESULTS

### Anti-oestrogen-resistant cell lines are more sensitive towards treatment with the vitamin D analogue EB1089

Dose–response experiments with MCF-7 cells and the two resistant cell lines clearly show that EB1089 are more potent for inhibition of growth of the resistant cell lines (Figure 1). The $IC_{50}$ values for MCF-7/TAM$^{R}$-1 and MCF-7/182$^{R}$-6 are $1.5 \times 10^{-9}$ M, and $1.5 \times 10^{-8}$ M for MCF-7, showing that EB1089 exerts a 10 times more potent growth inhibitory effect on the anti-oestrogen resistant cell lines.

### The response rate towards treatment with EB1089 correlates with the Bcl-2 expression level

The response towards EB1089 has recently been shown to correlate with the Bcl-2 protein level in a MCF-7 subline (Mathiasen et al, 1999). We therefore tested whether there were any differences in the basal Bcl-2 expression level among the cell lines, and whether EB1089 had effects on Bcl-2 expression. Figure 2 shows that the basal expression level was 6–8 fold higher in MCF-7 cells compared to both anti-oestrogen resistant cell lines. The MCF-7/TAM$^{R}$-1 and MCF-7/182$^{R}$-6 resistant sublines have almost the same basal Bcl-2 expression level. EB1089 alone did not show any regulatory effects on Bcl-2 protein expression, in the concentration range $10^{-11}$ M to $10^{-7}$ M (Figure 2).

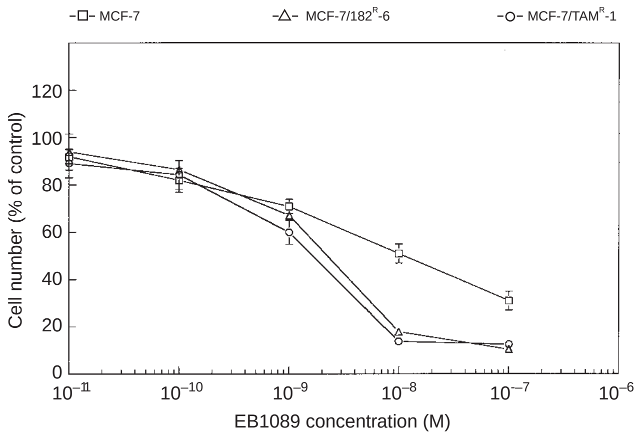

**Figure 1** Dose–response curves for effect of EB1089 on cell proliferation of MCF-7, MCF-7/TAM$^{R}$-1 and MCF-7/182$^{R}$-6 cells. MCF-7, MCF-7/TAM$^{R}$-1 and MCF-7/182$^{R}$-6 cells were grown 1 week in 1% FCS before onset of experiment. Cells ($2 \times 10^{4}$) were seeded in each 2 $cm^2$ multi-well, and 2 days after seeding experimental media with the indicated concentrations of EB1089 were added. Medium was renewed every 2nd or 3rd day. Cell numbers in quadruplicate wells were determined 6 days after addition of experimental medium. Mean values (expressed as percentage of the corresponding control culture without EB1089) and SDs are shown. SDs are not displayed when lower than 3%. The results are from one representative experiment out of 3 independent experiments with reproducible results

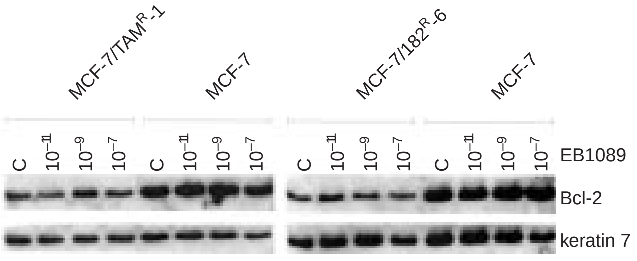

**Figure 2** Western blot (ECL) showing the expression of Bcl-2 in MCF-7, MCF-7/ TAM$^{R}$-1 and MCF-7/182$^{R}$-6 cells. Bcl-2 expression was determined after 6 days of hormonal treatment. Total cell extracts (30 μg of protein) from the 3 cell lines were loaded in each lane on a 15% SDS-PAGE gel, electroblotted, and immunostained with a primary mouse monoclonal antibody against human Bcl-2. The blot was stripped and immunostained with a primary mouse monoclonal antibody against human Keratin 7 as a control for protein loading. The immunocomplexes were visualized using the ECL$^{PLUS}$ detection system (Amersham Pharmacia Biotech). C: Control 1% FCS; $10^{-11}$ M, $10^{-9}$ M and $10^{-7}$ M EB1089. Representative results from one of three independent experiments are shown

### Oestradiol reduces the growth inhibitory response to EB1089 treatment concomitant with an increase in Bcl-2 expression

It has been shown that oestradiol induces Bcl-2 expression at the transcriptional level in MCF-7 cells, and protects the cells from induction of apoptosis (Dong et al, 1999; Mathiasen et al, 1999; Perillo et al, 2000). We therefore tested whether oestradiol could abolish or reduce the growth inhibitory effect of EB1089. Oestradiol almost completely abolished the growth inhibitory effect of EB1089 on MCF-7, exerted a partial abrogation in

© 2001 Cancer Research Campaign 

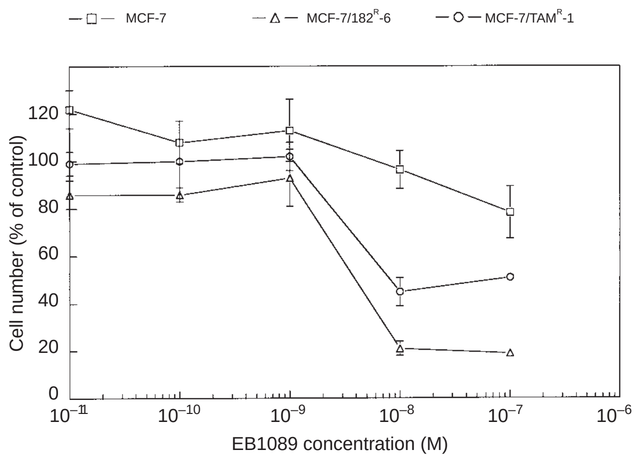

**Figure 3** Dose–response curves for effect of EB1089 plus oestradiol on cell proliferation of MCF-7, MCF-7/TAM$^R$-1 and MCF-7/182$^R$-6 cells. MCF-7, MCF-7/TAM$^R$-1 and MCF-7/182$^R$-6 cells were grown 1 week in 1% FCS before onset of experiment. Cells ($2 \times 10^4$) were seeded in each 2 $cm^2$ multi-well, and 2 days after seeding experimental media with the indicated concentrations of EB1089 plus $10^{-9}$ M oestradiol were added. Medium was renewed every 2nd or 3rd day. Cell numbers in quadruplicate wells were determined 6 days after addition of experimental medium. Mean values (expressed as percentage of the corresponding control culture without hormone) and SDs are shown. SDs are not displayed when lower than 3%. The results are from one representative experiment out of 3 independent experiments with reproducible results

MCF-7/TAM$^R$-1, and had no effect on MCF-7/182$^R$-6 cells (Figure 3 in comparison with Figure 1).

As observed in Figure 3, oestradiol did not abrogate the effect of EB1089 to the same extent in the 3 cell lines, and we tested whether the induction of Bcl-2 by oestradiol alone or in combination with EB1089 was different among the cell lines. Oestradiol alone induced the Bcl-2 protein expression 1.5–1.7 fold in MCF-7, 2.5–3.4 fold in MCF-7/TAM$^R$-1, and 2–2.5 fold in MCF-7/182$^R$-6 cells (Figure 4A). Although, the relative fold induction of Bcl-2 by oestradiol in the resistant cell lines was higher than in MCF-7 cells, the level never reached the same high amount as found in MCF-7 cells (Figure 4A). The induction of Bcl-2 by oestradiol in combination with EB1089 was 1.3–1.6 fold in MCF-7 and 1.5–2 fold in MCF-7/TAM$^R$-1, whereas in MCF-7/182$^R$-6 cells no induction could be detected (Figure 4B).

### EB1089 down-regulates the oestrogen receptor α

We speculated whether the ERα level could be important for the reduced oestradiol induction of Bcl-2 in combination with EB1089. Figure 4C shows that the ERα is down regulated by $10^{-7}$ M EB1089, the same concentration reducing the Bcl-2 induction in combination with oestradiol observed in the anti-oestrogen resistant cell lines (Figure 4B, lanes 6 and 9). In MCF-7 cells the ERα level is reduced about 40–50% by EB1089, in MCF-7/TAM$^R$-1 cells there is a 65–70% reduction, and in MCF-7/182$^R$-6 the ERα is completely down-regulated, suggesting that the reduced induction of Bcl-2 by oestradiol in combination with EB1089 is due to the down-regulation of ERα by EB1089.

### DISCUSSION

The data presented in this work show that anti-oestrogen-resistant cell lines are 10 times more sensitive towards treatment with the vitamin D analogue EB1089 than parent MCF-7 cells. We also show that the resistant cell lines have a lower basal Bcl-2 expression level than parent MCF-7 cells. This is in concert with our previous observation that all the resistant cell lines have a lower basal expression level of both ERα and oestrogen-regulated genes. Furthermore, they all lack PR expression (Lykkesfeldt et al, 1994, 1995). Several clinical studies have shown that the Bcl-2 expression in breast tumours is strongly correlated to both ERα and PR expression, and Bcl-2 expression is an indicator of a favourable outcome following endocrine treatment (Gee et al, 1994; Lipponen et al, 1995; Silvestrini et al, 1996; Elledge et al, 1997; Keen et al, 1997; Kobayashi et al, 1997; Olopade et al, 1997; Holmqvist et al, 1999). The decreased Bcl-2 expression associated with decreased ERα expression, and lack of response to anti-oestrogens observed in the resistant cell lines is in concert with the above-mentioned clinical studies.

The ratio between the pro-apoptotic and anti-apoptotic members in the Bcl-2-family has been shown to dictate the sensitivity or resistance of cells to a wide variety of apoptotic stimuli (Oltvai and Korsmeyer, 1994). In the two resistant cell lines the basal expression level of Bcl-2 is 6–8 fold lower than in the MCF-7 cell line, and consequently the critical ratio may be shifted in the pro-apoptotic direction rendering these cells more sensitive to apoptosis induced by EB1089, compared to parent MCF-7 cells. In MCF-7 cells, the critical ratio of Bcl-2 required to inhibit apoptosis may be reached at a 1.3–1.6 fold induction by oestradiol as indicated by the ability of oestradiol to abrogate the inhibitory effect of EB1089 in these cells. The induction of Bcl-2 with oestradiol in combination with EB1089 is 2.5–3.4 fold in MCF-7/TAM$^R$-1 cells. However, this Bcl-2 level is significantly lower than in oestradiol-treated MCF-7 cells and may, therefore, not be sufficient to protect the cells completely against the EB1089-induced apoptotic stimuli. Further support for the association between the Bcl-2 expression level and the effect of EB1089 is obtained by our results with the MCF-7/182$^R$-6 cells, in which oestradiol in combination with EB1089 neither induces Bcl-2 expression nor abolishes growth inhibition. A similar association between the Bcl-2 expression level and the response to EB1089 treatment has recently been observed in a different model system with Bcl-2-transfected cell lines (Mathiasen et al, 1999). The reduced induction of Bcl-2 by oestradiol in combination with EB1089 can be explained by the EB1089 mediated down-regulation of the ERα which is required for oestradiol induction of Bcl-2. EB1089-mediated down-regulation of ERα has also been reported by others (Simboli et al, 1997; Swami et al, 2000).

We did not observe any EB1089 regulation of the Bcl-2 expression (Figure 2), indicating that EB1089 does not mediate its effect via a down-regulation of this anti-apoptotic protein in our model system. This is in contrast to what has been shown by others, who found an EB1089 mediated down-regulation of Bcl-2 (Simboli et al, 1997). However, not only Bcl-2 but also IGF-binding proteins (IGFBPs) may be important for growth inhibition.

It has been reported that both EB1089 and the anti-oestrogens tamoxifen and ICI 182 780 up-regulate IGFBP-3 and IGFBP-5 in MCF-7 cells, and suggested that both EB1089 and the anti-oestrogens mediate their growth inhibitory effect via up-regulation of IGFBP-3 and IGFBP-5 (Rozen et al, 1997; Colston et al, 1998), which are known to induce apoptosis in MCF-7 cells (Huynh et al, 1996a, 1996b). Our data showing the lack of cross-resistance between tamoxifen, ICI 182 780 and EB1089 reported in this study and in Lykkesfeldt et al (1995) indicate that different mechanisms are responsible for growth inhibition mediated by these

© 2001 Cancer Research Campaign

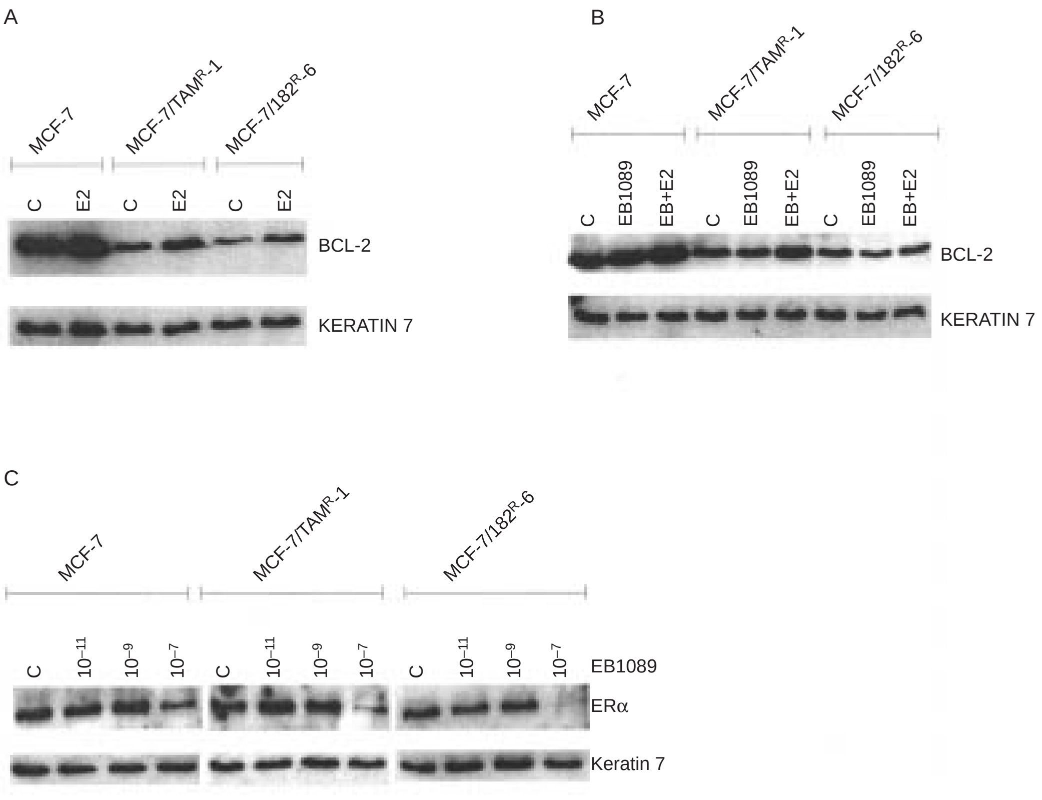

**Figure 4** Western blot (ECL) showing Bcl-2 and ERα expression in MCF-7, MCF-7/TAM$^R$-1 and MCF-7/182$^R$-6 cells treated with EB 1089 and oestradiol. Bcl-2 and ERα expression was determined after 6 days of hormonal treatment. Total cell extracts (30 µg of protein) were loaded in each lane on a 15% SDS-PAGE gel, electroblotted, and probed with a primary mouse monoclonal antibody against human Bcl-2, or a primary mouse monoclonal anti-human ERα antibody. The blots were stripped and immunostained with a primary mouse monoclonal antibody against human keratin 7 as a control for protein loading. The immunocomplexes were visualized using the ECL$^{PLUS}$ detection system (Amersham Pharmacia Biotech). Expression of Bcl-2. (**A**) C: Control 1% FCS; E2: $10^{-9}$ M 17-β-oestradiol. Expression of Bcl-2, (**B**) C: Control 1% FCS, EB: $10^{-7}$ M EB1089 and EB+E2: $10^{-7}$ M EB1089 plus $10^{-9}$ M 17-β-oestradiol. Expression of ERα. (**C**) C: Control 1% FCS; $10^{-11}$ M, $10^{-9}$ M and $10^{-7}$ M EB 1089. Representative results from one of the three independent experiments are shown drugs. At present, we are exploring whether IGFBP-3 and IGFBP-5 are involved in growth inhibition. In accordance with our assumption, JoEllen Welsh has also suggested that vitamin $D_3$ compounds and anti-oestrogens trigger growth arrest and apoptosis in MCF-7 cells by distinct mechanisms (Welsh, 1997). The JoEllen Welsh group has shown that MCF-7 cells resistant towards vitamin $D_3$ are sensitive to tamoxifen and ICI 182 780 treatment (Welsh, 1994; Narvaez and Welsh, 1997; Nolan et al, 1998).

Importantly, our data strongly suggest the Bcl-2 protein is not involved in development of resistance towards anti-oestrogens, due to the 6–8 fold lower Bcl-2 expression compared to parent MCF-7 cells. Furthermore our data demonstrate that acquired resistance towards anti-oestrogens does not render the cells multi-resistant to induction of apoptosis by other agents like EB1089.

Our experiments indicate that there may be a positive correlation between the degree of hormone independence and the response rate toward treatment with EB1089. MCF-7 cells are totally oestrogen dependent, MCF-7/TAM$^R$-1 are slightly oestrogen independent and MCF-7/182$^R$-6 are almost completely oestrogen independent for growth in vitro and in vivo (Lykkesfeldt et al, 1994; Jensen et al, 1999; Larsen et al, 1999). We have shown here for the first time, that it is favourable, with respect to treatment with EB1089, that the anti-oestrogen-resistant cells have progressed into a less hormone-dependent state, with a reduced expression of oestrogen-regulated genes e.g. Bcl-2, due to a lower ERα content.

Furthermore, the fact that both anti-oestrogen-resistant cell lines are more sensitive towards EB1089 than the parent MCF-7 cells looks promising for future treatment strategies. Clinical studies using EB1089 as treatment of different cancer types have demonstrated that the drug has no severe hypocalcaemia effects in humans (Gulliford et al, 1998; El Abdaimi et al, 1999). It has recently been reported that the development of Seocalcitol™ is active as an anti-cancer drug (Hansen et al, 2000). We therefore suggest that Seocalcitol™ (EB1089) may prove useful in treatment of breast cancer patients who have relapsed on anti-oestrogen treatment, and that the Bcl-2 expression level may be used to select the patients most likely to respond to treatment with Seocalcitol™.

### ACKNOWLEDGEMENTS

This work was supported by grants from the Danish Cancer Society. The secretarial help of Ms P Riis Kofoed-Hansen is highly appreciated. We thank Mogens W Madsen and Lise Binderup LEO Pharmaceuticals for providing us with EB1089.

*© 2001 Cancer Research Campaign* 

### REFERENCES

Brünner N, Boysen B, Jirus S, Skaar TC, Holst HC, Lippman J, Frandsen T, Spang Thomsen M, Fuqua SW and Clarke R (1997) MCF7/LCC9: An antiestrogen-resistant MCF-7 variant in which acquired resistance to the steroidal antiestrogen ICI 182,780 confers an early cross-resistance to the nonsteroidal antiestrogen tamoxifen. *Cancer Res* **57**: 3486–3493

Colston KW, Perks CM, Xie SP and Holly JM (1998) Growth inhibition of both MCF-7 and Hs578T human breast cancer cell lines by vitamin D analogues is associated with increased expression of insulin-like growth factor binding protein-3. *J Mol Endocrinol* **20**: 157–162

Dong LA, Wang WL, Wang F, Stoner M, Reed JC, Harigai M, Samudio I, Kladde MP, Vyhlidal C and Safe S (1999) Mechanisms of transcriptional activation of bcl-2 gene expression by 17 beta-oestradiol in breast cancer cells. *J Biol Chem* **274**: 32099–32107

El Abdaimi K, Papavasiliou V, Rabbani SA, Rhim JS, Goltzman D and Kremer R (1999) Reversal of hypercalcemia with the vitamin D analogue EB1089 in a human model of squamous cancer. *Cancer Res* **59**: 3325–3328

Elledge RM, Green S, Howes L, Clark GM, Berardo M, Allred DC, Pugh R, Ciocca D, Ravdin P, O'Sullivan J, Rivkin S, Martino S and Osborne CK (1997) bcl-2, p53, and response to tamoxifen in estrogen receptor-positive metastatic breast cancer: a Southwest Oncology Group study. *J Clin Oncol* **15**: 1916–1922

Gee JM, Robertson JF, Ellis IO, Willsher P, McClelland RA, Hoyle HB, Kyme SR, Finlay P, Blamey RW and Nicholson RI (1994) Immunocytochemical localization of BCL-2 protein in human breast cancers and its relationship to a series of prognostic markers and response to endocrine therapy. *Int J Cancer* **59**: 619–628

Gulliford T, English J, Colston KW, Menday P, Moller S and Coombes RC (1998) A phase I study of the vitamin D analogue EB 1089 in patients with advanced breast and colorectal cancer. *Br J Cancer* **78**: 6–13

Hansen CM, Hamberg KJ, Binderup E and Binderup L (2000) Seocalcitol (EB 1089): A vitamin D analogue of anti-cancer potential. Background, design, synthesis, pre-clinical and clinical evaluation. *Curr Pharm Des* **6**: 803–828

Holmqvist P, Lundstrom M and Stal O (1999) Apoptosis and Bcl-2 expression in relation to age, tumor characteristics and prognosis in breast cancer. South-East Sweden Breast Cancer Group. *Int J Biol Markers* **14**: 84–91

Howell A, Dodwell DJ, Laidlaw I, Anderson H and Anderson E (1990) Tamoxifen as an agonist for metastatic breast cancer. In: *Endocrine Therapy of Breast Cancer IV* Veronesi (ed), pp. 49–58. Springer Verlag: Berlin

Howell A, Defriend D, Robertson J, Blamey R and Walton P (1995) Response to a specific antioestrogen (ICI 182780) in tamoxifen-resistant breast cancer. *Lancet* 29–30

Huynh H, Yang X and Pollak M (1996a) Estradiol and antiestrogens regulate a growth inhibitory insulin-like growth factor binding protein 3 autocrine loop in human breast cancer cells. *J Biol Chem* **271**: 1016–1021

Huynh H, Yang XF and Pollak M (1996b) A role for insulin-like growth factor binding protein 5 in the antiproliferative action of the antiestrogen ICI 182780. *Cell Growth Differ* **7**: 1501–1506

James SY, Mercer E, Brady M, Binderup L and Colston KW (1998a) EB1089, a synthetic analogue of vitamin D, induces apoptosis in breast cancer cells in vivo and in vitro. *Br J Pharmacol* **125**: 953–962

Jensen BL, Skouv J, Lundholt BK and Lykkesfeldt AE (1999) Differential regulation of specific genes in MCF-7 and the ICI 182780-resistant cell line MCF-7/182$^R$-6. *Br J Cancer* **79**: 386–392

Keen JC, Dixon JM, Miller EP, Cameron DA, Chetty U, Hanby A, Bellamy C and Miller WR (1997) The expression of Ki-Sl and BCL-2 and the response to primary tamoxifen therapy in elderly patients with breast cancer. *Breast Cancer Res Treat* **44**: 123–133

Kobayashi S, Iwase H, Ito Y, Yamashita H, Iwata H, Yamashita T, Ito K, Toyama T, Nakamura T and Masaoka A (1997) Clinical significance of bcl-2 gene expression in human breast cancer tissues. *Breast Cancer Res Treat* **42**: 173–181

Larsen SS, Madsen MW, Jensen BL and Lykkesfeldt AE (1997) Resistance of human breast-cancer cells to the pure steroidal anti-oestrogen ICI 182,780 is not associated with a general loss of estrogen-receptor expression or lack of estrogen responsiveness. *Int J Cancer* **72**: 1129–1136

Larsen SS, Egeblad M, Jäätelä M and Lykkesfeldt AE (1999) Acquired antiestrogen resistance in MCF-7 human breast cancer sublines is not accomplished by altered expression of receptors in the ErbB-family. *Breast Cancer Res Treat* **58**: 41–56

Lipponen P, Pietilainen T, Kosma VM, Aaltomaa S, Eskelinen M and Syrjanen K (1995) Apoptosis suppressing protein bcl-2 is expressed in well-differentiated breast carcinomas with favourable prognosis. *J Pathol* **177**: 49–55

Love SC, Gibson DF, Ratnam AV and Bikle DD (1996) Antiestrogen potentiation of antiproliferative effects of vitamin D3 analogues in breast cancer cells. *Cancer Res* **56**: 2789–2794

Lykkesfeldt AE and Briand P (1986) Indirect mechanism of oestradiol stimulation of cell proliferation of human breast cancer cell lines. *Br J Cancer* **53**: 29–35

Lykkesfeldt AE and Sørensen EK (1992) Effect of estrogen and antiestrogens on cell proliferation and synthesis of secreted proteins in the human breast cancer cell line MCF-7 and a tamoxifen resistant variant subline, AL-1. *Acta Oncol* **31**: 131–138

Lykkesfeldt AE, Madsen MW and Briand P (1994) Altered expression of estrogen-regulated genes in a tamoxifen-resistant and ICI 164,384 and ICI 182,780 sensitive human breast cancer cell line, MCF-7/TAMR-1. *Cancer Res* **54**: 1587–1595

Lykkesfeldt AE, Larsen SS and Briand P (1995) Human breast cancer cell lines resistant to pure anti-estrogens are sensitive to tamoxifen treatment. *Int J Cancer* **61**: 529–534

Madsen MW, Reiter BE, Larsen SS, Briand P and Lykkesfeldt AE (1997) Estrogen receptor messenger RNA splice variants are not involved in antiestrogen resistance in sublines of MCF-7 human breast cancer cells. *Cancer Res* **57**: 585–589

Mathiasen IS, Colston KW and Binderup L (1993) EB 1089, a novel vitamin D analogue, has strong antiproliferative and differentiation inducing effects on cancer cells. *J Steroid Biochem Mol Biol* **46**: 365–371

Mathiasen IS, Lademann U and Jaattela M (1999) Apoptosis induced by vitamin D compounds in breast cancer cells is inhibited by Bcl-2 but does not involve known caspases or p53. *Cancer Res* **59**: 4848–4856

Narvaez CJ and Welsh J (1997) Differential effects of 1,25-dihydroxyvitamin D3 and tetradecanoylphorbol acetate on cell cycle and apoptosis of MCF-7 cells and a vitamin D3-resistant variant. *Endocrinology* **138**: 4690–4698

Nolan E, Donepudi M, Van Weelden K, Flanagan L and Welsh J (1998) Dissociation of vitamin D-3 and anti-estrogen mediated growth regulation in MCF-7 breast cancer cells. *Mol Cell Biochem* **188**: 13–20

Olopade OI, Adeyanju MO, Safa AR, Hagos F, Mick R, Thompson CB and Recant WM (1997) Overexpression of BCL-x protein in primary breast cancer is associated with high tumor grade and nodal metastases. *Cancer J Sci Am* **3**: 230–237

Oltvai ZN and Korsmeyer SJ (1994) Checkpoints of dueling dimers foil death wishes. *Cell* **79**: 189–192

Osborne CK, Yochmowitz MG, Knight WA and McGuire WL (1980) The value of estrogen and progesterone receptors in the treatment of breast cancer. *Cancer* **46**: 2884–2888

Osborne CK, Coronado HE, Hilsenbeck SG, McCue BL, Wakeling AE, McClelland RA, Manning DL and Nicholson RI (1995) Comparison of the effects of a pure steroidal antiestrogen with those of tamoxifen in a model of human breast cancer. *J Natl Cancer Inst* **87**: 746–750

Perillo B, Sasso A, Abbondanza C and Palumbo G (2000) 17beta-Estradiol inhibits apoptosis in MCF-7 cells, inducing bcl-2 expression via two estrogen-responsive elements present in the coding sequence. *Molecular and Cellular Biology* **20**: 2890–2901

Rozen F, Yang XF, Huynh H and Pollak M (1997) Antiproliferative action of vitamin D-related compounds and insulin-like growth factor-binding protein 5 accumulation. *J Natl Cancer Inst* **89**: 652–656

Silvestrini R, Benini E, Veneroni S, Daidone MG, Tomasic G, Squicciarini P and Salvadori B (1996) p53 and bcl-2 expression correlates with clinical outcome in a series of node-positive breast cancer patients. *J Clin Oncol* **14**: 1604–1610

Simboli CM, Narvaez CJ, van WK, Tenniswood M and Welsh J (1997) Comparative effects of 1,25(OH)2D3 and EB1089 on cell cycle kinetics and apoptosis in MCF-7 breast cancer cells. *Breast Cancer Res Treat* **42**: 31–41

Swami S, Krishnan AV and Feldman D (2000) 1alpha,25-Dihydroxyvitamin D3 down-regulates estrogen receptor abundance and suppresses estrogen actions in MCF-7 human breast cancer cells. *Clin Cancer Res* **6**: 3371–3379

Welsh J (1994) Induction of apoptosis in breast cancer cells in response to vitamin D and antiestrogens. *Biochem Cell Biol* **72**: 537–545

Welsh J (1997) Vitamin D compounds as potential therapeutics for estrogen-independent breast cancer [editorial]. *Nutrition* **13**: 915–917

Wiseman LR, Johnson MD, Wakeling AE, Lykkesfeldt AE, May FE and Westley BR (1993) Type I IGF receptor and acquired tamoxifen resistance in oestrogen-responsive human breast cancer cells. *Eur J Cancer* **29A**: 2256–2264

Xie SP, James SY and Colston KW (1997) Vitamin D derivatives inhibit the mitogenic effects of IGF-I on MCF-7 human breast cancer cells. *J Endocrinol* **154**: 495–504

Xie SP, Pirianov G and Colston KW (1999) Vitamin D analogues suppress IGF-I signalling and promote apoptosis in breast cancer cells. *Eur J Cancer* **35**: 1717–1723

 *© 2001 Cancer Research Campaign*

## References

[PDF_00547] Brünner N., Boysen B., Jirus S., Skaar T. C., Holst-Hansen C., Lippman J., Frandsen T., Spang-Thomsen M., Fuqua S. A., Clarke R. (1997). MCF7/LCC9: an antiestrogen-resistant MCF-7 variant in which acquired resistance to the steroidal antiestrogen ICI 182,780 confers an early cross-resistance to the nonsteroidal antiestrogen tamoxifen.. Cancer Res.

[PDF_00552] Colston K. W., Perks C. M., Xie S. P., Holly J. M. (1998). Growth inhibition of both MCF-7 and Hs578T human breast cancer cell lines by vitamin D analogues is associated with increased expression of insulin-like growth factor binding protein-3.. J Mol Endocrinol.

[PDF_00556] Dong L., Wang W., Wang F., Stoner M., Reed J. C., Harigai M., Samudio I., Kladde M. P., Vyhlidal C., Safe S. (1999). Mechanisms of transcriptional activation of bcl-2 gene expression by 17beta-estradiol in breast cancer cells.. J Biol Chem.

[PDF_00560] El Abdaimi K., Papavasiliou V., Rabbani S. A., Rhim J. S., Goltzman D., Kremer R. (1999). Reversal of hypercalcemia with the vitamin D analogue EB1089 in a human model of squamous cancer.. Cancer Res.

[PDF_00563] Elledge R. M., Green S., Howes L., Clark G. M., Berardo M., Allred D. C., Pugh R., Ciocca D., Ravdin P., O'Sullivan J. (1997). bcl-2, p53, and response to tamoxifen in estrogen receptor-positive metastatic breast cancer: a Southwest Oncology Group study.. J Clin Oncol.

[PDF_00568] Gee J. M., Robertson J. F., Ellis I. O., Willsher P., McClelland R. A., Hoyle H. B., Kyme S. R., Finlay P., Blamey R. W., Nicholson R. I. (1994). Immunocytochemical localization of BCL-2 protein in human breast cancers and its relationship to a series of prognostic markers and response to endocrine therapy.. Int J Cancer.

[PDF_00573] Gulliford T., English J., Colston K. W., Menday P., Moller S., Coombes R. C. (1998). A phase I study of the vitamin D analogue EB 1089 in patients with advanced breast and colorectal cancer.. Br J Cancer.

[PDF_00576] Hansen C. M., Hamberg K. J., Binderup E., Binderup L. (2000). Seocalcitol (EB 1089): a vitamin D analogue of anti-cancer potential. Background, design, synthesis, pre-clinical and clinical evaluation.. Curr Pharm Des.

[PDF_00579] Holmqvist P., Lundström M., Stål O. (1999). Apoptosis and Bcl-2 expression in relation to age, tumor characteristics and prognosis in breast cancer. South-East Sweden Breast Cancer Group.. Int J Biol Markers.

[PDF_00591] Huynh H., Yang X. F., Pollak M. (1996). A role for insulin-like growth factor binding protein 5 in the antiproliferative action of the antiestrogen ICI 182780.. Cell Growth Differ.

[PDF_00594] James S. Y., Mercer E., Brady M., Binderup L., Colston K. W. (1998). EB1089, a synthetic analogue of vitamin D, induces apoptosis in breast cancer cells in vivo and in vitro.. Br J Pharmacol.

[PDF_00597] Jensen B. L., Skouv J., Lundholt B. K., Lykkesfeldt A. E. (1999). Differential regulation of specific genes in MCF-7 and the ICI 182780-resistant cell line MCF-7/182R-6.. Br J Cancer.

[PDF_00601] Keen J. C., Dixon J. M., Miller E. P., Cameron D. A., Chetty U., Hanby A., Bellamy C., Miller W. R. (1997). The expression of Ki-S1 and BCL-2 and the response to primary tamoxifen therapy in elderly patients with breast cancer.. Breast Cancer Res Treat.

[PDF_00605] Kobayashi S., Iwase H., Ito Y., Yamashita H., Iwata H., Yamashita T., Ito K., Toyama T., Nakamura T., Masaoka A. (1997). Clinical significance of bcl-2 gene expression in human breast cancer tissues.. Breast Cancer Res Treat.

[PDF_00613] Larsen S. S., Egeblad M., Jättelä M., Lykkesfeldt A. E. (1999). Acquired antiestrogen resistance in MCF-7 human breast cancer sublines is not accomplished by altered expression of receptors in the ErbB-family.. Breast Cancer Res Treat.

[PDF_00609] Larsen S. S., Madsen M. W., Jensen B. L., Lykkesfeldt A. E. (1997). Resistance of human breast-cancer cells to the pure steroidal anti-estrogen ICI 182,780 is not associated with a general loss of estrogen-receptor expression or lack of estrogen responsiveness.. Int J Cancer.

[PDF_00617] Lipponen P., Pietiläinen T., Kosma V. M., Aaltomaa S., Eskelinen M., Syrjänen K. (1995). Apoptosis suppressing protein bcl-2 is expressed in well-differentiated breast carcinomas with favourable prognosis.. J Pathol.

[PDF_00620] Love-Schimenti C. D., Gibson D. F., Ratnam A. V., Bikle D. D. (1996). Antiestrogen potentiation of antiproliferative effects of vitamin D3 analogues in breast cancer cells.. Cancer Res.

[PDF_00623] Lykkesfeldt A. E., Briand P. (1986). Indirect mechanism of oestradiol stimulation of cell proliferation of human breast cancer cell lines.. Br J Cancer.

[PDF_00633] Lykkesfeldt A. E., Larsen S. S., Briand P. (1995). Human breast cancer cell lines resistant to pure anti-estrogens are sensitive to tamoxifen treatment.. Int J Cancer.

[PDF_00629] Lykkesfeldt A. E., Madsen M. W., Briand P. (1994). Altered expression of estrogen-regulated genes in a tamoxifen-resistant and ICI 164,384 and ICI 182,780 sensitive human breast cancer cell line, MCF-7/TAMR-1.. Cancer Res.

[PDF_00625] Lykkesfeldt A. E., Sørensen E. K. (1992). Effect of estrogen and antiestrogens on cell proliferation and synthesis of secreted proteins in the human breast cancer cell line MCF-7 and a tamoxifen resistant variant subline, AL-1.. Acta Oncol.

[PDF_00636] Madsen M. W., Reiter B. E., Larsen S. S., Briand P., Lykkesfeldt A. E. (1997). Estrogen receptor messenger RNA splice variants are not involved in antiestrogen resistance in sublines of MCF-7 human breast cancer cells.. Cancer Res.

[PDF_00640] Mathiasen I. S., Colston K. W., Binderup L. (1993). EB 1089, a novel vitamin D analogue, has strong antiproliferative and differentiation inducing effects on cancer cells.. J Steroid Biochem Mol Biol.

[PDF_00643] Mathiasen I. S., Lademann U., Jättelä M. (1999). Apoptosis induced by vitamin D compounds in breast cancer cells is inhibited by Bcl-2 but does not involve known caspases or p53.. Cancer Res.

[PDF_00646] Narvaez C. J., Welsh J. (1997). Differential effects of 1,25-dihydroxyvitamin D3 and tetradecanoylphorbol acetate on cell cycle and apoptosis of MCF-7 cells and a vitamin D3-resistant variant.. Endocrinology.

[PDF_00649] Nolan E., Donepudi M., VanWeelden K., Flanagan L., Welsh J. (1998). Dissociation of vitamin D3 and anti-estrogen mediated growth regulation in MCF-7 breast cancer cells.. Mol Cell Biochem.

[PDF_00652] Olopade O. I., Adeyanju M. O., Safa A. R., Hagos F., Mick R., Thompson C. B., Recant W. M. (1997). Overexpression of BCL-x protein in primary breast cancer is associated with high tumor grade and nodal metastases.. Cancer J Sci Am.

[PDF_00656] Oltvai Z. N., Korsmeyer S. J. (1994). Checkpoints of dueling dimers foil death wishes.. Cell.

[PDF_00661] Osborne C. K., Coronado-Heinsohn E. B., Hilsenbeck S. G., McCue B. L., Wakeling A. E., McClelland R. A., Manning D. L., Nicholson R. I. (1995). Comparison of the effects of a pure steroidal antiestrogen with those of tamoxifen in a model of human breast cancer.. J Natl Cancer Inst.

[PDF_00658] Osborne C. K., Yochmowitz M. G., Knight W. A., McGuire W. L. (1980). The value of estrogen and progesterone receptors in the treatment of breast cancer.. Cancer.

[PDF_00665] Perillo B., Sasso A., Abbondanza C., Palumbo G. (2000). 17beta-estradiol inhibits apoptosis in MCF-7 cells, inducing bcl-2 expression via two estrogen-responsive elements present in the coding sequence.. Mol Cell Biol.

[PDF_00669] Rozen F., Yang X. F., Huynh H., Pollak M. (1997). Antiproliferative action of vitamin D-related compounds and insulin-like growth factor-binding protein 5 accumulation.. J Natl Cancer Inst.

[PDF_00672] Silvestrini R., Benini E., Veneroni S., Daidone M. G., Tomasic G., Squicciarini P., Salvadori B. (1996). p53 and bcl-2 expression correlates with clinical outcome in a series of node-positive breast cancer patients.. J Clin Oncol.

[PDF_00675] Simboli-Campbell M., Narvaez C. J., van Weelden K., Tenniswood M., Welsh J. (1997). Comparative effects of 1,25(OH)2D3 and EB1089 on cell cycle kinetics and apoptosis in MCF-7 breast cancer cells.. Breast Cancer Res Treat.

[PDF_00678] Swami S., Krishnan A. V., Feldman D. (2000). 1alpha,25-Dihydroxyvitamin D3 down-regulates estrogen receptor abundance and suppresses estrogen actions in MCF-7 human breast cancer cells.. Clin Cancer Res.

[PDF_00681] Welsh J. (1994). Induction of apoptosis in breast cancer cells in response to vitamin D and antiestrogens.. Biochem Cell Biol.

[PDF_00683] Welsh J. (1997). Vitamin D compounds as potential therapeutics for estrogen-independent breast cancer.. Nutrition.

[PDF_00685] Wiseman L. R., Johnson M. D., Wakeling A. E., Lykkesfeldt A. E., May F. E., Westley B. R. (1993). Type I IGF receptor and acquired tamoxifen resistance in oestrogen-responsive human breast cancer cells.. Eur J Cancer.

[PDF_00688] Xie S. P., James S. Y., Colston K. W. (1997). Vitamin D derivatives inhibit the mitogenic effects of IGF-I on MCF-7 human breast cancer cells.. J Endocrinol.

[PDF_00691] Xie S. P., Pirianov G., Colston K. W. (1999). Vitamin D analogues suppress IGF-I signalling and promote apoptosis in breast cancer cells.. Eur J Cancer.

